# The role of TIM3^+^ NK and TIM3^-^ NK cells in the immune pathogenesis of severe aplastic anemia

**DOI:** 10.2478/jtim-2023-0104

**Published:** 2024-03-21

**Authors:** Shaoxue Ding, Tian Zhang, Yingying Lei, Chunyan Liu, Zhaoyun Liu, Rong Fu

**Affiliations:** Department of Hematology, Tianjin Medical University General Hospital, Tianjin 300052, China

**Keywords:** aplastic anemia, natural killer cells, TIM3, immune, pathogenesis

## Abstract

**Background:**

Natural killer (NK) cells play important immunoregulatory roles in the immune pathogenesis of severe aplastic anemia (SAA). Our previous research showed that SAA caused a decrease in T cell immunoglobulin mucin-3 (TIM3) expression on NK cells. Here we investigated the expression of surface receptors, and the cytotoxicity of peripheral TIM3^+^ NK and TIM3^-^ NK cells in patients with SAA.

**Methods:**

The expressions of surface receptors and cytoplasmic protein of TIM3^+^ NK and TIM3^-^ NK cells from peripheral blood were detected by FCM. The functions of mDCs, and apoptosis rate of K562 cells after co-culture with TIM3^+^ NK and TIM3^-^ NK cells were maesured by FCM. Westren-blot was used to detect the changes of TIM3^+^ NK and TIM3^-^ NK signaling pathway proteins (AKT, P-AKT) and compare the functional activity of the two groups.

**Results:**

Activating receptors NKG2D and Granzyme B were higher, while inhibiting receptors NKG2A, CD158a and CD158b were lower on TIM3^-^ NK cells compared with TIM3^+^ NK cells in patients with SAA. In SAA, the expression of CD80 and CD86 on mDCs (Myeloid dendritic cells) was significantly decreased after incubation with TIM3^-^ NK cells. The apoptosis rate (AR) of K562 cells was significantly increased after being incubated with TIM3^-^ NK cells in SAA. The level of signal pathway protein AKT of TIM3^-^ NK cells in SAA was similar to that of TIM3^+^ NK cells, and the levels of P-AKT and P-AKT/AKT ratio of TIM3^-^ NK cells were significantly higher than those of TIM3^+^ NK cells.

**Conclusions:**

Therefore, TIM3 exerts its inhibitory effect on NK cells and participates in the immune pathogenesis of SAA. Low expression of TIM3 contributes to the enhancement of NK cell activity which in turn inhibits the immune activation state of SAA and improves the disease state. Our research may aid the development of new therapeutic strategies based on TIM3-NK cells infusion for the treatment of SAA.

## Introduction

Aplastic anemia (AA) is a bone marrow failure (BMF) disorder, resulting in bone marrow hypocellularity and peripheral pancytopenia. Severe AA (SAA) is a subtype of this disease characterized by very low bone marrow cellularity of less than 25%, with significant morbidity and mortality; some immune mechanisms play a key role in its pathogenesis.^[1,2]^ Patients with AA are treated with either immunosuppressive therapy (IST) using anti-thymocyte globulin (ATG) and cyclosporine (CsA) or hematopoietic stem cell transplantation (HSCT), if a matched donor is available.^[3]^ Although the pathophysiology of AA remains elusive, in patients with the most common form of acquired AA, the myeloid dendritic cells (mDCs) induce naïve T cells differentiation into Th1 cells by secreting interleukin-2 (IL-2), while the autologous T lymphocytes suppress replicative activity and induce hematopoietic stem and progenitor cells (HSPCs) apoptosis.^[4,5]^ In addition, scanty CD4^+^CD25^+^Foxp3^+^ regulatory T cells are found in most patients with AA, which may contribute to autologous T lymphocytes generation and AA development.^[5,6]^

In patients with AA, oligoclonal T cells in marrow were detected to show mature memory/effector phenotypes, implicating memory T cells might participate in the pathophysiological process of AA.^[7]^ Interferon-γ (IFN-γ) has been implicated in SAA in humans, although the underlying mechanisms driving hematopoietic failure are unknown and may involve macrophages acting as sensors for IFN-γ. The reduction of macrophages rescued thrombocytopenia, increased bone marrow megakaryocytes, preserved platelet primer stem cells, and increased the ability of platelet regeneration of transplanted hematopoietic stem cells. In a mouse model of SAA, IFN-γ specifically maintains macrophages and is associated with loss of platelet-biased hematopoietic stem cells, severe thrombocytopenia, and death, whereas targeting macrophages attenuates disease and promotes survival.^[8]^

Natural killer (NK) cells are large granular lymphocytes that can directly lyse target cells without prior sensitization and produce cytokine and chemokines in the early phase of an immune response, bridging the gap between innate and adaptive immunities.^[9]^ Previous studies have elucidated the hypofunction mechanism of NK cells in some autoimmune diseases, which primarily involve inhibitory and activation receptors on cell membranes.^[10,11]^ We have previously investigated the peripheral circulating NK cells in patients with SAA, which is significantly decreased, as well as NKp46 activating receptors and perforin’s cytotoxic factor when compared with healthy controls.^[12,13]^ However, the role of the over-active NK cells in SAA remains unclear.

TIM3 is regarded as a negative regulator in Th1 immunity, which is deemed as a contributor to effector T cell exhaustion. Several studies suggested that TIM3 may serve as a marker of NK cell activation and maturation, as it is widely detected on NK cell membranes.^[14]^ Previously, we have found that the expression of TIM3 on NK cells and CD56^dim^ NK subsets are lower in newly-diagnosed SAA patients compared with healthy controls, and it negatively correlated with the severity of pancytopenia; in SAA patients, the expression of TIM3 recovered to normal level after IST.^[15]^ It is hypothesized that a low expression of TIM3 on NK cells might contribute to NK cell dysfunction and the subsequent progression of bone marrow failure in SAA.^[15]^

However, the specific fluctuation of TIM3^+^ NK and TIM3^-^ NK cells has not been analyzed. In this study, we further tried to elucidate the functional changes and explore the roles of TIM3^+^ NK and TIM3^-^ NK cells in SAA. We will provide new therapeutic ideas to improve the efficacy of SAA treatment further.

## Methods

### Patients

After approval by the Ethics Committee of the Tianjin Medical University, we acquired informed written consent from all patients or their parents in accordance with the Declaration of Helsinki. The study enrolled a total of 51 individuals, including 18 newly diagnosed and 18 remission SAA (R-SAA) patients from the Hematology Department of the Tianjin Medical University General Hospital, and 15 healthy controls (HC), from January 2020 to January 2021. The laboratory characteristics of the subjects are shown in Table 1.

**Table 1:** Characteristics of patients with SAA and Healthy controls

Covariates	SAA (*n* = 18)	R-SAA (*n* = 18)	Healthy control (*n* = 15)	*P* value
Median age at diagnosis (range), years	39 (14-79)	42 (18-75)	48 (17-61)	0.15
Gender, male/female	9/9	10/8	8/7	0.52
Severity of AA, *n*. patients VSAA/SAA	4/14	4/14	-	-
Initital Neut count × 10^9^/L	0.64 ± 0.22	2.74 ± 0.47	2.94 ± 0.44	-
Initital RBC, count × 10^12^//L	2.48 (1.88, 2.85)	4.26 (3.99, 5.04)	4.44 (4.08, 5.12)	-
Initital HB, g/L	76.00 ± 6.58	134.00 ± 7.94	142.00 ± 8.07	-
Initital PLT count × 10^9^/L	16.00 ± 7.93	240.50 ± 56.38	261.00 ± 52.07	-
Initital RET count × 10^9^/L	11.60 ± 5.24	64.31 ± 15.22	60.39 ± 14.17	-
Abnormal chromosome	Absence	Absence	Absence	-

SAA: severe aplastic anemia; VSAA: very severe aplastic anemia; Neut: neutrophils; RBC: Red blood cell; HB: hemoglobin; PLT: platelets; RET: reticulocytes.

In order to explore whether blood counts affect the expression level of TIM-3 in NK cells of SAA patients, clinical indicators and NK cell TIM-3 expression levels of 18 SAA patients were analyzed by Pearson correlation analysis. The results showed that the expression rate of TIM-3 in NK cells of 18 SAA patients had no correlation with the proportion of peripheral blood Ret%, hemoglobin level, platelet counts and granulocyte counts (Supplementary Figure 1).

For newly diagnosed SAA patients, blood samples were collected before they received any treatment; for the other participants, blood samples were collected upon enrollment. All blood samples were tested to exclude autoimmune diseases, cancer biomarkers, or infections. All patients were screened for chromosome abnormalities and PNH clones. We did not detect any abnormal chromosomes or PNH clones in any patient.

The diagnosis of SAA was established according to the international AA Study Group Criteria.^[16]^ Patients who suffered from SAA and achieved complete remission after IST containing rATG (rabbit ATG) plus CsA were regarded as R-SAA patients, which is defined as normal complete blood count and transfusion-independence.^[16]^

### Flow cytometry antibodies

The conjugated antibodies used for functional molecules detection were the following: PerCP-Cy5.5 Mouse Anti-Human CD3 (catalog: 560835), FITC Mouse Anti-Human CD16 (catalog: 561308), PE Mouse Anti-Human NKp46 (catalog: 557991), PE Mouse Anti-Human NKp44 (catalog: 558563), PE Mouse Anti-Human NKG2A (catalog: 555889), PE Mouse Anti-Human CD158a (catalog: 556063), PE Mouse Anti-Human CD158b (catalog: 559785), PE Mouse Anti-Human Perforin (catalog: 556437), PE Mouse Anti-Human Granzyme B (catalog: 561142), APC Mouse Anti-Human CD11c (catalog: 559877), PE Mouse Anti-Human CD80 (catalog: 557227), PE Mouse Anti-Human CD86 (catalog: 555658), PE-Cy7 Mouse Anti-Human CD56 (catalog: 557747), Mouse Anti-Human CD86 (catalog: 555658) and FITC Annexin V Apoptosis Detection Kit (catalog: 556570) were purchased from BD Pharmingen (Franklin Lakes, USA). PE Mouse Anti-Human CD314 (NKG2D)(catalog: 12-5879-42), APC Mouse Anti-Human TIM3 (catalog: 17-3109-42), PerCP Mouse Anti-Human HLA-DR (catalog: MA1-10348) were purchased from Invitrogen (Carlsbad, USA).

### Measurement of TIM3^+^ and TIM3^-^ NK cells function from peripheral blood

A series of conjugated antibodies or their matched control isotypes were added to fresh peripheral blood in varying volumes as recommended by the manufacturer and mixed at 4 °C in the dark for 30 min in TrueCount tubes (BD Biosciences, Franklin Lakes, NJ, USA). For the detection of surface receptors, conjugated antibodies against NKG2A, NKG2D, CD158a (KIR2DL1), CD158b (KIR2DL2), NKp46, NKp44, along with CD3, CD56 and TIM3, or their matched control isotypes were used. After incubation, cells were lysed using FACS RBC lysing solution (BD Biosciences, Franklin Lakes, NJ, USA), followed by two washes in Phosphate Buffer Solution (PBS). For perforin and granzyme B intracellular staining, the cells were mixed with 1.0 mL of FACSTM permeabilizing solution (BD) before staining with perforin and granzyme B. The analysis was done using a Beckman CytoFLEX Flow Cytometer. Data were analyzed using Kaluza v2.0 (Coulter). The controls used for photo multiplier tube (PMT) voltage setting or compensation, and gating strategy with gate boundaries, or negative/positive controls used for some analysis strictly follow the operation process.

### Isolation and purification of TIM3^+^ and TIM3^-^ NK cells

**Figure 1:**
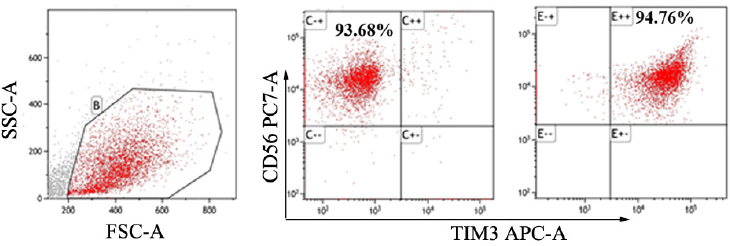
solation and purification of TIM3^+^ and TIM3^-^ NK cells by fetal calf serum (FCS).

Peripheral blood mononuclear cells (PBMNCs) were isolated from peripheral blood using density gradient centrifugation with Ficoll-Paque Plus solution (Amersham Bioscience, Uppsala, Sweden) and then stained with conjugated CD3, CD56, and TIM3 antibodies. After washing with PBS, highly purified and sterile TIM3^+^ NK and TIM3^-^ NK cell populations were obtained using FACSAriaII (BD Biosciences, Franklin Lakes, NJ, USA) according to different surface staining (CD3^-^CD56^+^TIM3^-^ and CD3^-^CD56^+^TIM3^+^). The purity of TIM3^+^ NK and TIM3^-^ NK cells were 90% and 95%, respectively (Figure 1).

### Adherent culture of mDCs

**Figure 2:**
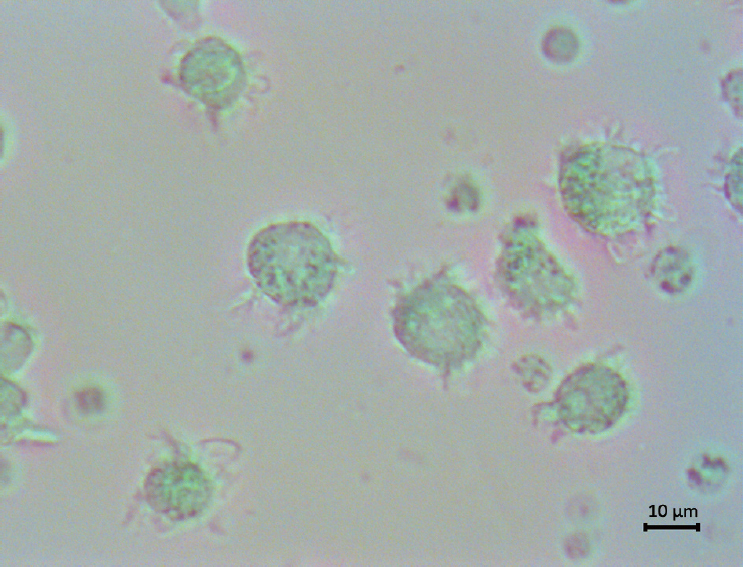
After 7 days of adherent culture and induction using rhGM-CSF and rhIL-4, mDCs were observed under an inverted microscope. Irregular protrusions similar to pseudopodia can be seen on these cells, which were in suspension.

Bone marrow aspirations were performed in each patient; we collected sterile bone marrow for BMMNCs isolation. No infection was observed and no transfusions were given 3 days prior. The isolated BMMNCs were plated in a RPMI 1640 culture medium containing 10% Fetal Bovine Serum (FBS) and 1% mycillin (Gibco BRL, Grand Island, NY, USA), and incubated for 2 h. Non-adherent cells were disposed and the adherent cells were treated with complete media containing 100 μg/L rhGM-CSF and 20 μg/L rhIL-4 (PeproTech Inc, USA) at 37°C in a 5% CO_2_-containing atmosphere. Fresh medium, rhGM-CSF, and rhIL-4 were added on day 3 and rhTNFα (1000 μg/mL) (PeproTech Inc.) on day 6. On day 7, we collected and counted mDCs from the supernatant ^[17,18]^ (Figure 2).

### Identification and sorting of mDCs

**Figure 3:**
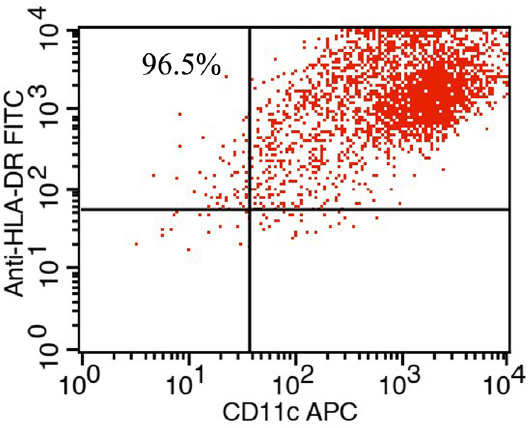
The purity of CD11c^+^HLA-DR^+^ cells obtained by fetal calf serum (FCS) was above 90%.

The collected mDCs were analyzed using flow cytometry with a combination of anti-HLA-DR-PerCP and anti-CD11c-APC mAbs. HLA-DR^+^CD11c^+^ cells were sorted and collected using a FacsAria flow cytometer. Flow cytometry data acquisition and analysis were carried out using the Cell Quest software, version 3.1 (Becton Dickinson). The purity of the mDC cells was 90%–95% (Figure 3).

### The effect of TIM3^+^ NK and TIM3^-^ NK cells on mdcs’ function

Sorted mDCs were cultured as described above. We then separated TIM3^+^ and TIM3^-^ NK cells from SAA patients using the FACSAriaII sorting system. The purified TIM3^+^ and TIM3^-^ NK cells were incubated with mDCs (mDC+TIM3^+^NK and mDC+TIM3^-^NK groups, respectively) at 37 °C with 5% CO_2_ at an effector-to-target ratio of 1: 1 for 48 h. The mDC cells incubated without NK cells in the culture medium acted as control (mDC_alone_ group). The expression of CD80 and CD86 on mDCs in the three groups were measured using FACS.

### Cytotoxic activity of TIM3^+^ NK and TIM3^-^ NK cells against K562 cells

The cytotoxic activity of NK cells was measured using FACS, co-cultured with K562 cells (Sigma-Aldrich, St Louis, USA). The purified TIM3^+^ and TIM3^-^ NK cells with K562 cells (K562+TIM3^+^ NK and K562+TIM3^-^ NK groups) were incubated at 37 °C with 5% CO_2_ at an effector-to-target ratio of 1: 1 for 48 h and then stained with FITC-Annexin V and Propidium Iodide (PI) for FACS detection. A sample of only K562 cells (K562_alone_ group) was used as a control. The cytotoxic activity of TIM3^+^ and TIM3^-^ NK cells was evaluated based on specific K562 cell apoptosis rates.

### Expression of post-receptor signaling pathway proteins of TIM3^+^ and TIM3^-^ NK cells

Isolated TIM3^+^ and TIM3^-^ NK cells were collected and lysed directly in RIPA buffer supplemented with complete protease (Roche, Basel, Switzerland) and phosphatase inhibitors (Solarbio Science & Technology, Beijing, China). Protein levels in the lysates were quantified using a BCA kit. Proteins were separated using 4%–20% Precast-Gel and transferred to nitrocellulose (NC) membranes (Pall Corporation, New York, NY, USA). The membranes were blocked with 10% skimmed milk (Chuntest Biotechnology, Shanghai, China) and subsequently incubated with anti-AKT, anti-Phospho-AKT, and anti-GAPDH antibodies (CST, Danvers, MA, USA) at a 1: 1000dilution. The antibodies were dissolved in a solution containing 5% dried milk in Tris-buffered saline with TBS-T (20 mmol/L Tris-HCl buffer, pH7.4, 150 mmol/L NaCl, 0.05% Tween 20). After extensive washing with PBS, the membranes were incubated with relevant horseradish peroxidase-conjugated secondary antibodies (1: 5000 dilution; CST). The labeled protein bands were detected using Super ECL Plus Detection Reagent. All protein levels were normalized to GAPDH.

### Statistical analysis

Data from at least three independent experiments are presented as mean ± SD. Significant differences between means were determined with multiple t-tests using the Holm-Sidak method and one-way ANOVA when a Gaussian distribution was assumed, and with a Kruskal-Wallis test when Gaussian distribution was not assumed. Multiple comparison tests were performed to compare particular pairs of control and patient groups. Statistical analysis was performed using GraphPad Prism version 6.0 and SPSS 21.0. *P* values < 0.05 were considered as statistically difference.

## Results

### TIM3^-^ NK cells in SAA, R-SAA, and healthy controls expressed higher activating receptors and lower inhibitory receptors than TIM3^+^ NK cells

**Figure 4:**
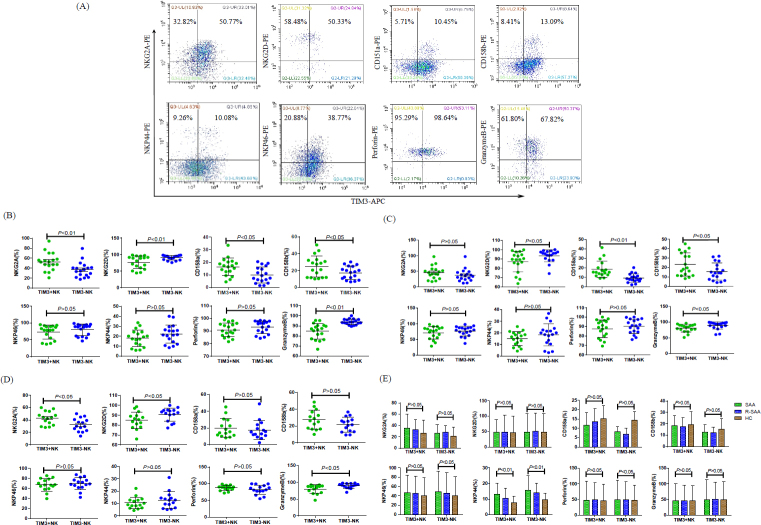
Measurement of the functional molecules of TIM3^+^ and TIM3^-^NK cells from peripheral blood. (A) Expression of NKG2A, NKG2D, CD158a, CD158b, NKp46, NKp44, Perforin, and GranzymeB on TIM3^-^NK and TIM3^+^NK cells in newly diagnosed SAA patients. (B) TIM3^-^NK cells expressed more NKG2D and Granzyme B than TIM3^+^NK cells. NKG2A, CD158a, and CD158b on TIM3^-^NK cell surface were lower than TIM3^+^NK cells in SAA. (C) TIM3^-^NK cells expressed higher NKG2D and Granzyme B, and lower CD158a and CD158b than TIM3^+^NK cells in R-SAA. (D) TIM3^-^NK cells expressed higher NKG2D and Granzyme B, and lower NKG2A than TIM3^+^NK cells in HC. (E) The expression of NKP44 on TIM3^+^NK cells in SAA groups was higher than that of HC groups, while the expression of NKP44, NKP46, and perforin in TIM3^-^NK cells was higher than that of HC groups.

We compared the expression of activating receptors (NKG2D, NKp46, NKp44) and inhibitory receptors (NKG2A, CD158a, and CD158b) on TIM3^+^ and TIM3^-^ NK cells from newly diagnosed SAA patients (Figure 4, A and B). The expression of NKG2D was significantly increased in TIM3^-^ NK cells compared with TIM3^+^ NK cells (*P* = 0.007). However, no differences were found in the expression of NKp46 and NKp44 between TIM3^-^ NK and TIM3^-^ NK cells. TIM3^-^ NK cells expressed less NKG2A, CD158a, and CD158b inhibitory receptors compared with TIM3^+^ NK cells (all *P*< 0.05). As for intracellular protein, TIM3^-^ NK cells expressed more granzyme B (*P* = 0.024). However, no statistical differences in perforin expression between TIM3^-^ NK and TIM3^+^ NK cells were found (*P* > 0.05).

As shown in R-SAA patients (Figure 4C), we found that activating receptor NKG2D increased in TIM3^-^ NK cells (*P* = 0.018), but we did not find any differences in the expression of NKp46 and NKp44 (all *P* > 0.05) between TIM3^-^ NK and TIM3^-^ NK cells. TIM3^-^ NK cells expressed less CD158a and CD158b inhibitory receptors, compared with TIM3^+^ NK cells (all *P* < 0.05). There were no significant differences in NKG2A expression between TIM3^-^ NK and TIM3^+^ NK cells (*P* > 0.05). The expression of intracellular granzyme B in TIM3^-^ NK cells was higher than that in TIM3^+^ NK cells (*P* = 0.011).

In healthy controls, the expression of NKG2D and Granzyme B on TIM3^-^ NK cells was higher than on TIM3^+^ NK cells (Figure 4D). As for the expression of inhibitory receptors, NKG2A on TIM3^-^ NK cells surface was lower than on TIM3^+^ NK cells. We did not find any differences in the expression of CD158a, CD158b, NKp46, NKp44, and perforin between TIM3^-^ NK and TIM3^+^ NK cells in healthy controls.

We also compared the functional molecules of TIM3^-^ NK and TIM3^+^ NK cells in SAA, R-SAA, and healthy control groups. As shown in Figure 4E, the expression of NKP44 on TIM3^+^ NK cells in SAA groups was higher than that of healthy control groups, while the expression of NKP44, NKP46, and perforin in TIM3^-^ NK cells was higher than that of healthy control groups. There were no significant differences in the expression of other functional molecules on TIM3^+^ NK and TIM3^-^ NK cells among the three groups.

### The expression of CD80 and CD86 on mDC cells was decreased after coculture with TIM3^+^ NK and TIM3^-^

**Figure 5:**
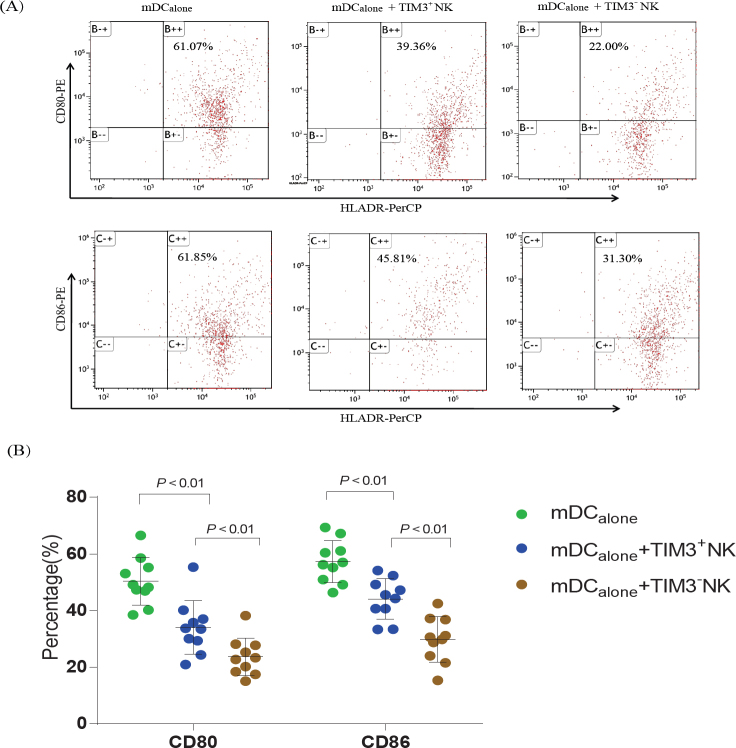
The effect of TIM3^+^ and TIM3^-^ NK cells on the function of mDCs. (A) Expression of CD80 and CD86 on mDC cells in the control, TIM3^+^ and TIM3^-^ NK groups in newly diagnosed SAA patients. (B) The expression of CD80 and CD86 was significantly decreased after incubation with TIM3^-^ and TIM3^+^ NK cells in SAA; especially in the TIM3^-^ NK group, it was significantly lower than that in the TIM3^+^NK groups (*P* < 0.01).

As shown in Figure 5 (A and B), in newly diagnosed SAA patients, the expression of surface proteins CD80 and CD86 on the mDC_alone_, mDC+TIM3^+^ NK, and mDC+TIM3^-^ NK groups was 50.39% ± 8.37%, 34.04% ± 9.46%, 23.69% ± 6.69%, and 57.32% ± 7.36%, 44.11% ± 7.16%, 29.81% ± 8.02%, respectively. After co-culture with NK cells from SAA patients, mDC expressed less CD80 and CD86, especially in the mDC+TIM3^-^ NK group, which was significantly lower than that in the mDC+TIM3^+^ NK group (*P* = 0.001).

### Comparison of cytotoxic activities of TIM3^+^ and TIM3^-^ NK cells in SAA

**Figure 6:**
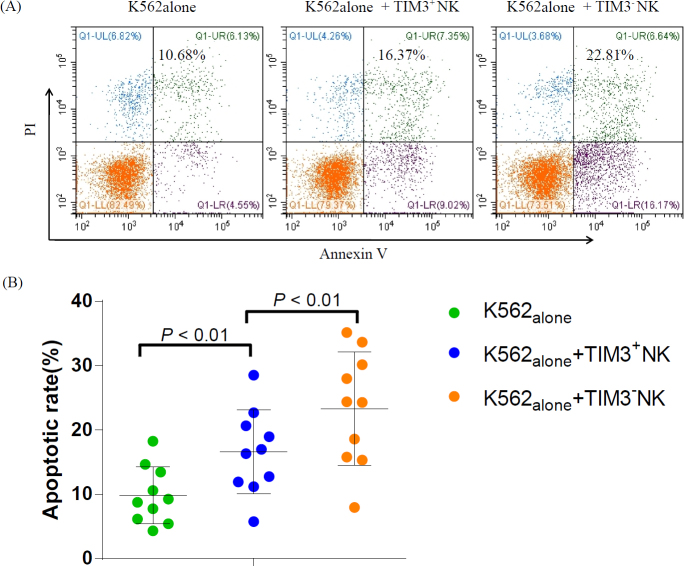
Comparison of cytotoxic activities of TIM3^+^ NK and TIM3^-^ NK cells in SAA. (A) AR of K562 cells in K562_alone_, K562+TIM3^+^ NK, and K562+TIM3^-^ NK groups. (B) The AR of K562 cells were significantly increased after incubation with TIM3^-^ NK and TIM3^+^ NK cells in SAA, especially those of the K562+TIM3^-^ NK group, which were significantly higher than those of the K562+TIM3^+^ NK groups (*P* < 0.01).

We investigated the cytotoxic activities of sorted TIM3^+^ NK and TIM3^-^ NK cells. We confirmed that both subsets of NK cells in SAA had cytotoxic activity. As shown in Figure 6 (A and B), the AR of K562 cells in K562_alone_, K562+TIM3^+^ NK, and K562+TIM3^-^ NK groups were 9.86% ± 4.43%, 16.57% ± 6.53%, and 23.32% ± 8.79%, respectively. The AR of K562 cells was significantly increased after incubation with TIM3^-^ and TIM3^+^NK cells in SAA, especially in the K562+TIM3^-^ NK group, which was significantly higher than that in the K562+TIM3^+^ NK group (*P* = 0.004).

### The expression of post-receptor signaling pathway proteins of TIM3^+^ and TIM3^-^ NK cells

**Figure 7:**
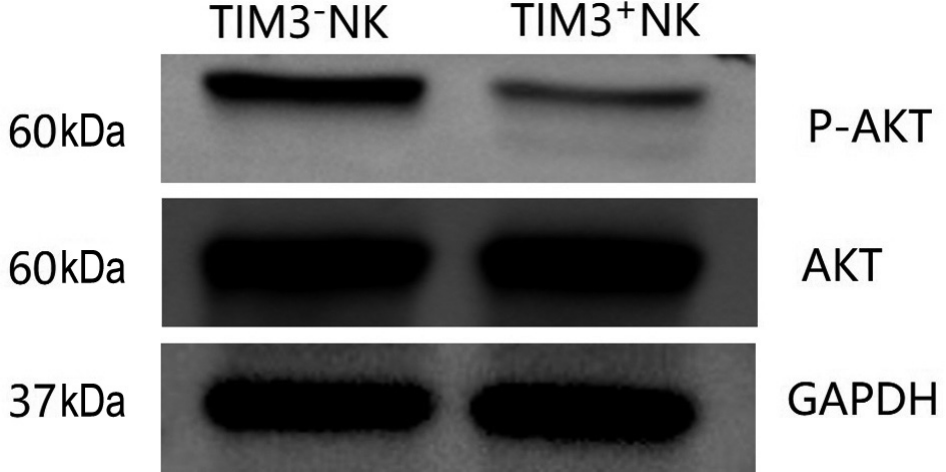
AKT and P-AKT levels in TIM3^-^NK and TIM3^+^NK cells in patients with SAA.

The results of the relative gray value analysis showed that the relative expression of AKT in TIM3^-^ NK and TIM3^+^ NK cells of the patients with SAA was 1.53 ± 0.22 and 1.48 ± 0.13, respectively, and the relative expression of P-AKT was 0.72 ± 0.11 and 0.28 ± 0.07 in TIM3^-^ NK and TIM3^+^ NK cells, respectively. The level of signal pathway protein AKT of TIM3^-^ NK cells in SAA was similar to that of TIM3^+^ NK cells, and the levels of P-AKT and P-AKT/ AKT ratio of TIM3^-^ NK cells were significantly higher than those of TIM3^+^ NK cells (*P* = 0.001). The biological activity of TIM3^-^ NK cells was higher than that of TIM3^+^ NK cells. TIM3 may play a role in inhibiting the activity of NK cells (Figure 7).

## Discussion

SAA is believed to be an autoimmune disease attributed to hematopoietic cell destruction by activated cytotoxic T lymphocytes (CTLs), causing HSPCs impairment through the Fas/FasL pathway and secreted perforin and granzyme B.^[19,20,21]^ NK cells are an important component of the innate immune system and play a core role in the regulation of adaptive immunity, which could inhibit antigen-presenting cells (APC), such as DCs and Tregs in patients with autoimmune diseases.^[22,23,24]^

Our previous studies have shown decreased NK cell proportions and overly-expressed activation receptors in newly diagnosed SAA patients, especially NKp46 and perforin.^[12,25]^ The cytotoxicity of SAA patients’ NK cells was enhanced, causing a higher apoptosis rate of K562 cells when co-cultured.^[26]^ All these results suggest that NK cells might constitute a negative regulatory factor to partially offset the T cell-mediated destruction of HSCs in SAA. CTL became overactive in patients with SAA induced by scanty and abnormal NK cells. Why is activating receptor expression of NK cells increased and its function enhanced in patients with SAA? Is the compensatory response due to the decrease in NK cells number, or are there other reasons? The reason why the function of NK cells is enhanced in patients with SAA is still unclear.

TIM3 is regarded as a negative regulator in Th1 immunity, which is deemed a contributor to effector T cell exhaustion.^[27]^ However, its role in NK cells is not clear. TIM3 is widely expressed on immune cells, such as monocytes, DCs, and NK cells, and is important in various immune responses, such as infection, autoimmunity, and tumor immunity. TIM3 also modulates the function of NK cells in many human diseases.^[28]^ TIM3 signaling blockade can increase the cytotoxicity and IFN-γ production of peripheral NK cells in patients with lung adenocarcinoma.^[29]^ In our previous study, we found that the expression of TIM3 on NK cells significantly decreased in newly diagnosed SAA patients, which was a potent cytotoxicity negative regulator of some immune cells.^[15]^ When stimulated by cytokines, TIM3 expression on NK cells increased, as well as IFN-γ. In addition, with disease progression, the expression and function of TIM3 changed.^[30,31]^

The lack of TIM3 disabled NK cells to negatively regulated cellular immunity in patients with SAA. It was hypothesized that scanty NK cells might fail to suppress the overactivity of mDCs and T cells, ultimately causing HSCs destruction in patients with SAA. The expression of TIM3 on NK cells is reduced and the function of NK cells is relatively enhanced; however, the function of the enhanced NK cell is not enough to offset the weakening of the immune surveillance caused by the decrease in the number of NK cells.^[15,26]^ Certainly, studies on different NK cell subsets in patients with SAA will be essential. Thus, we report on the function of TIM3^+^ NK and TIM3^-^ NK cells in SAA.

In the present study, we further demonstrated the role of TIM3^+^ NK and TIM3^-^ NK cells in SAA. The expression of activation marker NKG2D and granzyme B were higher in TIM3^-^ NK cells than that in TIM3^+^ NK cells, and the inhibitor markers NKG2A, CD158a, and CD158b were lower than those of TIM3^+^ NK cells in patients with SAA. Similar findings were obtained from healthy controls and R-SAA patients. The only difference was that the expression of NKG2A in TIM3^+^ NK cells in R-SAA patients and the expression of CD158a and CD158b in healthy controls were slightly higher than those of TIM3^-^ NK cells, but there was no significant difference between them.

We further found that the expression of CD80 and CD86 on mDCs was significantly decreased after incubation with TIM3^-^ and TIM3^+^ NK cells in SAA, while the expression of CD80 and CD86 on mDCs in the mDC+TIM3^-^ NK group was significantly lower than that in the mDC+TIM3^+^NK group. Thus, TIM3^-^ NK cells can suppress the function of mDC, and the inhibitory effect is stronger than that of TIM3^+^ NK cells. In patients with SAA, TIM3^-^ NK cells might be immature as TIM3 is a marker of NK cell maturity. TIM3^-^ NK cells are more cytotoxic in patients with SAA, as our previous in vitro studies have demonstrated.^[26]^ NK cells can inhibit the mDC in AA patients and TIM3 how to play this role? We will further explore this mechanism in future research.

In this study, we demonstrated that both subsets of NK cells had cytotoxic activity in patients with SAA. The K562 cell line (a human chronic myeloid leukemia carcinoma cell line), frequently used as a target cell in NK cytotoxicity assays.^[32]^ In this study, to assay the cytotoxicity of NK cells, K562 cells are used. The AR of K562 induced by TIM3^-^ NK cells isolated from SAA was higher than that of TIM3^+^ NK cells. The cytotoxicity of TIM3^-^ NK cells was stronger than that of TIM3^+^ NK cells in vitro. We did not find differences in cytotoxicity levels of TIM3^+^ NK cells between SAA and HC individuals. In addition, we found that the level of the signal pathway protein AKT of TIM3^-^ NK cells in SAA was similar to that of TIM3^+^ NK cells, and the levels of P-AKT and P-AKT/AKT ratio of TIM3^-^ NK cells were significantly higher than those of TIM3^+^ NK cells. These changes indicated that the function of TIM3^-^ NK cells was higher than that of TIM3^+^ NK cells in patients with SAA.

In patients with SAA, we previously found an increased number of mDC, which expressed more CD86. The imbalance of mDC subsets might promote Th0 cells to polarize to Th1 cells, which results in over production of autologous T lymphocytes and destruction of HSCs in SAA patients.^[2,33]^

The lack of NK cells might fail to suppress the function of DC and activated T cells. Thus, the number of NK cells recovered, especially TIM3^-^ NK cells, which can control abnormal autoimmune activity. These findings indicated that TIM3^-^ NK cells may be protective in SAA pathogenesis. By infusing TIM3^-^ NK cells into SAA patients, we can increase the number of NK cells and improve the treatment efficacy of SAA patients.

## Conclusions

The expression of TIM3 in NK cells was decreased in patients with SAA, and the function of TIM3^-^ NK cells was stronger than that of TIM3^+^ NK cells. In the immune pathogenesis of SAA, the expression of TIM3 on NK cells was reduced and the function of NK cells was relatively enhanced. However, the activity of the enhanced NK cells is not enough to offset the weakening of the immune surveillance caused by the decrease in NK cell numbers, which cannot effectively inhibit the activation of mDCs. In turn, mDCs cause the abnormal activation of the CD8^+^ T cells, resulting in the excessive apoptosis of the HSCs. Our research may aid the development of new therapeutic strategies based on TIM3^-^ NK cells infusion for the treatment of SAA.

## Supplementary Material

Supplementary Material
