# Supplementary material for: The role of TIM3+ NK and TIM3- NK cells in the immune pathogenesis of severe aplastic anemia
Source: J Transl Int Med. 2024 Mar 21;12(1):96–105. doi: 10.2478/jtim-2023-0104 (PMC10956726; doi:10.2478/jtim-2023-0104)
Supplement: Supplementary file 1 — Supplementary Material [file jtim-2023-0104_sm.pdf]

Supplementary Materials

The expression rate of TIM-3 in NK cells of SAA patients was correlated with clinical indicators

In order to explore whether blood counts affect the expression level of TIM-3 in NK cells of SAA patients, clinical indicators and NK cell TIM-3 expression levels of 18 SAA patients were analyzed by Pearson correlation analysis. The results showed that the expression rate of TIM-3 in NK cells of 18 SAA patients had no correlation with the proportion of peripheral blood Ret%, hemoglobin level, platelet counts and granulocyte counts (Supplementary Figure 1).

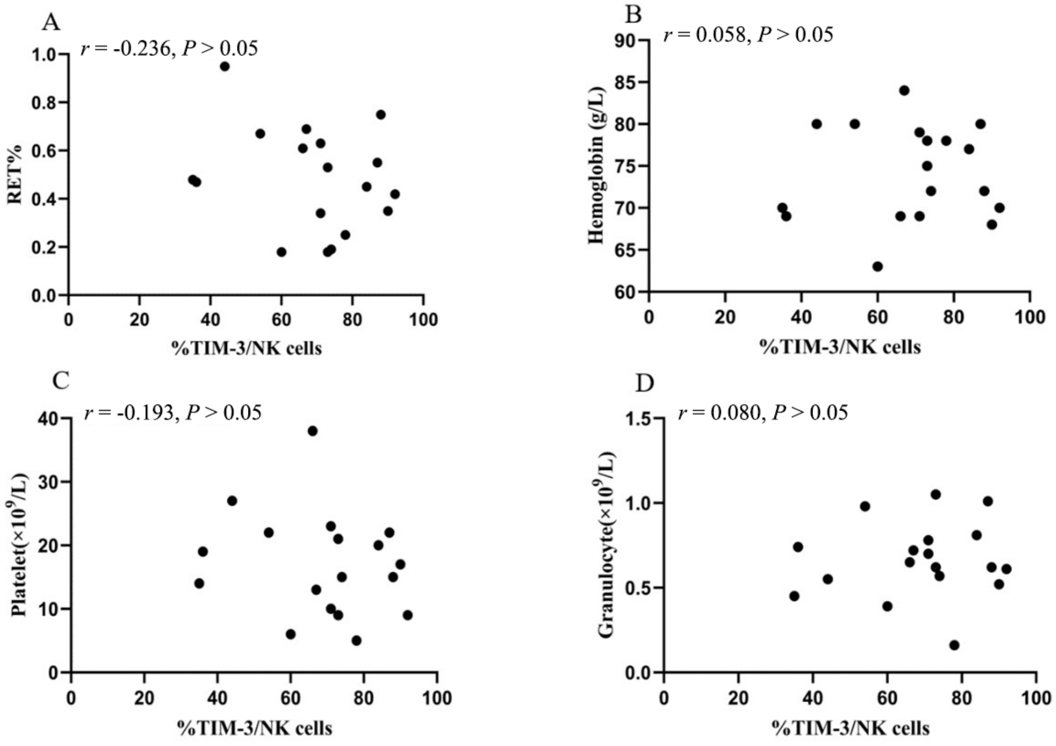

Supplementary Figure 1 The expression rate of TIM-3 in NK cells of SAA patients was correlated with clinical indicators

A: The expression rate of TIM-3 in NK cells of SAA patients had no correlation with RET% (r = -0.236, P > 0.05); B: The expression rate of TIM3 in NK cells of SAA patients had no correlation with the hemoglobin level (r = 0.058, P > 0.05); C: The expression rate of TIM3 in NK cells of SAA patients had no correlation with the platelet counts (r = -0.193, P > 0.05); D: The expression rate of TIM3 in NK cells of SAA patients had no correlation with the granulocyte counts (r = 0.080, P > 0.05).

We believe that there is no correlation between the blood indicators of SAA patients and the expression of TIM-3 in NK cells. Therefore, the influence of blood counts on TIM-3 expression, such as RET%, hemoglobin levels, platelet counts and granulocyte counts, can be excluded.
